# Fat Content Quantification with US Attenuation Coefficient: Phantom Correlation with MRI Proton Density Fat Fraction

**DOI:** 10.3390/diagnostics16010080

**Published:** 2025-12-25

**Authors:** Rongying Chen, Genglin Zhang, Jie Zeng, Yin Zhang, Haixin Chen, Jie Ren, Xin Chen, Manli Wu, Haoming Lin, Ting Zhang

**Affiliations:** 1 Department of Medical Ultrasonics, Third Affiliated Hospital of Sun Yat-Sen University, Guangzhou 510630, Chinazengjie5@mail.sysu.edu.cn (J.Z.);; 2 Department of Infectious Disease, Third Affiliated Hospital of Sun Yat-Sen University, Guangzhou 510630, China; 3 National-Regional Key Technology Engineering Laboratory for Medical Ultrasound, Guangdong Key Laboratory of Biomedical Measurements and Ultrasound Imaging, School of Biomedical Engineering, Shenzhen University Medical School, Shenzhen 518071, China

**Keywords:** attenuation coefficient, ultrasound, fatty liver, MRI-PDFF

## Abstract

**Objective:** The aim of this study was to evaluate the consistency and reproducibility of attenuation coefficient (AC) measurements using different commercial ultrasound (US) across via a phantom experiment to investigate the relationship between the AC and MRI-derived proton density fat fraction (MRI-PDFF) values and the conversion equation. **Methods:** Twelve phantoms containing varying fat proportions (0–100%) were constructed. Phantom ACs were estimated via three US attenuation systems, including attenuation imaging (ATI), ultrasound attenuation analysis (USAT), and the US-guided attenuation parameter (UGAP), along with MRI-PDFF. Agreement among the AC values from the three ultrasonic attenuation systems was evaluated. Linear correlation analysis was used to explore the ACs, fat concentrations of the phantom, and MRI-PDFF measurements, from which a linear conversion formula between the ultrasonic attenuation parameters and the MRI-PDFF was derived. **Results:** MRI-PDFF and phantom fat concentration measurements appeared with a strong linear correlation (R^2^ = 0.996, *p* < 0.001). For the three US attenuation parameters, both inter-operator and intra-operator intraclass correlation coefficients (ICCs) ranged from 0.990 to 0.995 and 0.989 to 0.995, respectively. Bland–Altman analysis revealed no significant differences between the above three (all *p* > 0.05). Significant linear relationships were demonstrated between ultrasound attenuation parameters and phantom fat concentration (r = 0.938–0.986; all *p* < 0.001), as well as between ultrasound attenuation parameters and MRI-PDFF values (r = 0.922–0.982; all *p* < 0.001). A conversion formula (fat proportions ≤ 50%) was derived: US (dB/cm/MHz) = 0.501 + 0.012 MRI-PDFF (%). **Conclusions:** AC across different commercial ultrasound devices demonstrated significant diagnostic value in fat concentrations that appeared good consistency in measuring phantom fat concentration both between and within groups. The linear relationship between AC and MRI-PDFF enables the application of a conversion formula.

## 1. Introduction

Metabolic dysfunction-associated steatotic liver disease (MASLD), previously termed metabolic-associated fatty liver disease (MAFLD) or nonalcoholic fatty liver disease (NAFLD), has emerged as the most prevalent chronic liver disease globally, affecting at least 30% of the adult population worldwide [1,2]. In China, the prevalence of NAFLD stands at 29.8%, making it, along with chronic viral hepatitis B or C, alcoholic fatty liver, and drug-induced liver injury, a primary cause of liver cirrhosis and liver cancer, a severe threat to the health of the Chinese population [3]. In 2023, it surpassed chronic hepatitis B as the most common chronic liver disease in China [4].

Steatosis is an initiating factor of MASLD, and the first diagnostic challenge is to accurately detect fat in the liver. Accurate quantification of liver fat content plays an important role in evaluating the risk of progression and liver-related events [2]. Currently, liver biopsy with histopathological examination is the gold standard for diagnosing fatty liver disease. The liver is classified based on the area of hepatocellular steatosis as follows: normal liver tissue S0 < 5%, mild fatty liver S1 5% to <33%, moderate fatty liver S2 33% to <66%, and severe fatty liver S3 ≥ 66%. However, this diagnostic method is invasive and carries risks of bleeding and infection [5,6]. MRI-based techniques for the quantitative assessment of hepatic steatosis have been recognized by multiple guidelines as the most accurate noninvasive method for quantifying liver fat content. Based on hepatic fat volume fraction, the liver can be classified as follows: mild fatty liver (<5%), moderate fatty liver (5% to <17%), and severe fatty liver (17% to <22%) [2]. However, owing to their high cost and venue constraints, both the European Association for the Study of the Liver (EASL) and American Association for the Study of Liver Diseases (AASLD) guidelines do not recommend the use of MRI-PDFF for screening in routine clinical settings [7,8]. However, conventional ultrasonography is less sensitive for detecting lower degrees of liver fat content and is limited by substantial interobserver variability [9].

Currently, the US attenuation-based fat quantification technique attenuation coefficient (AC) is widely used to evaluate the severity of MASLD and relies on the energy loss of the acoustic signals while traveling through the tissue [2]. The attenuation estimation techniques have been validated by using a phantom with known attenuation values and mathematical simulations [10]. However, AC measurements developed by different manufacturers using various acoustic attenuation measurement systems have inconsistent thresholds for diagnosing and grading fatty liver disease with low degrees of discrimination [11]. Moreover, there is a lack of data regarding the correlation of the ultrasound AC with the MRI-PDFF, which limits the use of the AC in clinical applications. A previous review noted that to better understand the variables affecting US attenuation measurements for the assessment of liver fat content, it is necessary to develop phantoms to compare various vendor systems [12,13]. Current research has introduced various fabrication approaches for multimodal hepatic steatosis phantoms. Madsen et al. incorporated graphite into gelatin–water–alcohol phantoms, where controlled graphite concentrations yield varying values in ultrasound attenuation coefficient measurements [14]. Zhao et al. developed an MRI phantom capable of simultaneous PDFF, R^2^*, and T1 modulation. They used peanut oil to modulate PDFF, MnCl_2_ and iron microspheres were used to modulate R^2^*, and NiCl_2_ was used to modulate the T_1_ of water (T_1,water_) [15] Chmarra et al. Mary developed a cost-effective, simple-to-fabricate multimodal liver phantom compatible with ultrasound, CT, and MRI. The phantom composition consists of six components: candle gel, Sephadex, agarose, glycerol, distilled water, and silicone string [16]. Endsley et al. developed an ultrasound phantom fabrication method that replicates fat vesicle sizes observed in MASLD, facilitating the validation of ultrasound quantification techniques [17].

This study employed ultrasound systems from three different manufacturers to measure attenuation coefficients, followed by consistency analysis of the results. The relationship between ultrasound attenuation coefficients and MRI-PDFF values was analyzed to derive a conversion formula for translating ultrasound-based fat quantification results into percentage values. The study aims to optimize the grading criteria for fatty liver disease in subsequent clinical research.

## 2. Materials and Methods

### 2.1. Phantom Preparation

The preparation of the fat phantom was improved upon the method described by Bush et al. [18]. A 3% agar powder was used as a gelling agent to create a solid-shaped phantom, while peanut oil served as the fat source. Additionally, 2% potassium sorbate was used as a preservative to extend the shelf life of the phantom, and 2% surfactant was used to emulsify the fat, ensuring a uniform distribution of fat in the water. Furthermore, 3% corn starch was added as a scatterer to increase the base attenuation value of the phantom.

The agar powder was used to facilitate phantom formation, while potassium sorbate and corn starch were employed to protect the phantoms from dissection. Both components were mixed to form an aqueous solution. The emulsifier was mixed with peanut oil to obtain an oil solution. The two solutions were mixed in the desired proportions, stirred via a magnetic stirrer, and heated on a hot plate until the mixture appeared uniformly milky white without visible phase separation. The hot plate was turned off, and stirring continued until the solution cooled. The mixture was then poured into pre-prepared containers, which were cylindrical plastic boxes (LOCK&LOCK, ROK, and HPL932D), with a large opening and sufficient depth to facilitate ultrasound data collection. The containers were shaken to remove air bubbles and then placed in a refrigerator at 4 °C for more than 10 h to allow the fat emulsion phantom to solidify completely. Phantoms with 12 different fat concentrations were prepared: 0%, 5%, 10%, 15%, 20%, 25%, 30%, 35%, 40%, 50%, 60%, and 100% (pure peanut oil).

### 2.2. MRI-PDFF Measurement

MRI-PDFF measurements were performed via a 3.0T MRI system (Discovery MR750, GE HealthCare, Waukesha, WI, USA). Axial breath-hold IDEAL-IQ sequences were acquired with the following parameters: TR = 3.7 ms, TE = 1.7 ms, slice thickness = 5.0 mm, bandwidth = 125 kHz, field of view (FOV) = 37 cm × 46 cm, matrix = 256 × 256, flip angle = 3°, and NEX = 1.00. After the phantoms were scanned via the PDFF (IDEAL-IQ sequence), the following images were automatically generated without offline processing: in-phase images, out-of-phase images, pure water images, pure fat images, fat fraction maps, and R^2^* relaxation rate maps. Fat fraction maps were used to directly measure the fat content of the phantoms. A senior radiologist placed 5 regions of interest (ROIs) at different levels of each phantom, avoiding container edges and impurities. The ROIs had an area of approximately 30–40 mm^2^. Five MRI-PDFF values were obtained, and the median value was recorded.

### 2.3. Acquisition of Attenuation Coefficient, Backscatter, and Speed of Sound

The US imaging was performed with a single Vantage 128TM Research Ultrasound Systems (Verasonics Inc., Kirkland, WA, USA) driving an L38-22 linear array transducer (Verasonics Inc., Kirkland, WA, USA) via the Verasonics adapter. The imaging process employed multi-angle compounded plane waves with an angular deflection range of ±4° (i.e., −4°, −2°, 0°, 2°, 4°). Radiofrequency (RF) data were acquired 4 times at each angle. The pulse repetition frequency (PRF) was set at 6.25 kHz, so the effective imaging frame rate during ULM data acquisition was 1250 Hz. The speed of sound sampling was achieved using a ring-array ultrasound transducer (Doppler Electronics Technology Co., Ltd., Guangzhou, China) with 256 elements and a diameter of 160 mm. Each element had a width of 0.5 mm, a height of 6 mm, a center frequency of 2 MHz, and a bandwidth of 70%. The transducer was immersed in a water bath and connected to a Verasonics ultrasound system for sampling. The algorithm was implemented with reference to previous literature using MATLAB R2024a [19,20].

### 2.4. Ultrasound Attenuation Coefficient Measurement

**Figure 1:**
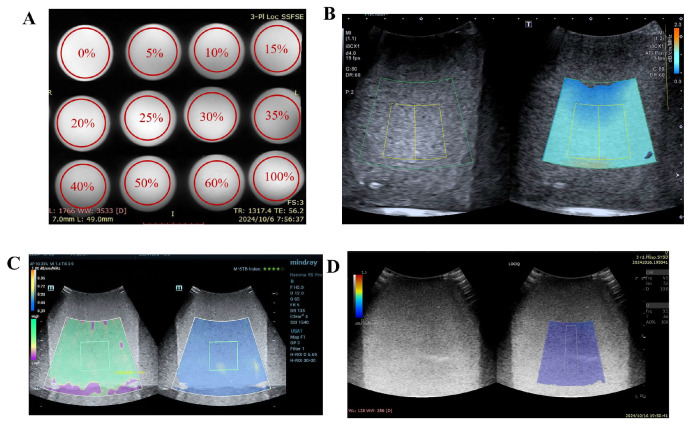
Examples of MRI-PDFF and ultrasound measurements of the phantom: (**A**) measurement example of MRI-PDFF; (**B**) measurement example of ATI; (**C**) measurement example of USAT; (**D**) measurement example of UGAP.

Only the authors with no conflicts of interest had full control of the inclusion of data or information. The fat phantoms were measured using three commercially available ultrasound attenuation measurement machines available at our institution, namely: attenuation imaging (ATI, Aplio 900, Canon, Tokyo, Japan), ultrasound attenuation imaging technology (USAT, Resona R9 Pro, Mindray, Shenzhen, China), and an ultrasound-guided attenuation parameter (UGAP, LOGIQ E11, GE HealthCare, Waukesha, WI, USA). The measured ultrasonic parameters are shown in Table 1. For each phantom, the cross-section was first displayed in B mode. Immediately after the conventional ultrasound procedure, attenuation coefficient examination was performed continuously by the same physician with the same scanner. The examination was performed at the location where the largest possible 2D view could be obtained. A sample box with the default setting was used to acquire the data from the three devices mentioned above. It was placed at least 1.5 cm below the phantom surface to avoid reverberation artifacts (Figure 1). Detailed information concerning attenuation imaging followed the previous guidelines [21,22,23]. For ATI measurements, the reliability of the results is displayed in terms of the R^2^ value. When R^2^ ≥ 0.8, good measurement stability is indicated. For the USAT measurements, the motion stability (M-STB) index and reliability map (RLB MAP) were used to evaluate the accuracy and stability of the measurements. Measurements were performed only when the M-STB index reached at least four stars and was displayed in green, and the RLB index exceeded 85%, indicating high reliability. For UGAP measurements, stability is automatically regulated by the machine, requiring no manual intervention.

The ultrasound measurements were performed by two sonographers with over ten years of experience. They were blinded to the fat concentration of the phantoms, AC measurement results from each other, and MRI-PDFF values of the phantoms. The order of operation between the two sonographers was randomized. Each ultrasonic attenuation parameter of each phantom was measured five times, and the interquartile range was recorded for analysis.

### 2.5. Statistical Analysis

The primary statistical hypotheses of this study were as follows: 1. There would be no significant difference in AC measurements among the three ultrasound devices (null hypothesis), versus the alternative that a significant difference exists. 2. There would be no significant linear association between ultrasound-derived AC and the reference standards (MRI-PDFF and phantom fat concentration gradient) from which a predictive formula could be derived (null hypothesis), versus the alternative that a significant association and viable predictive model exist. To test these hypotheses, all analyses were performed using SPSS 24.0 and MedCalc 23.0 software. Data normality was assessed using Shapiro–Wilk tests, percent–percent plots, and quantil–equantile plots. Differences in AC measurements among the three devices were evaluated using the Kruskal–Wallis test. Measurement agreement was assessed using the intraclass correlation coefficient (ICC), with values > 0.75 and >0.9 indicating good and excellent agreement, respectively [24], and Bland–Altman analysis was applied to evaluate agreement between different ultrasound parameters and inter-operator consistency. To investigate the association between AC and reference standards, the Wilcoxon matched-pairs signed-rank test was used to assess differences between MRI-PDFF and phantom concentration values, the Pearson correlation coefficient (r) was calculated to quantify linear associations, and linear regression was employed to derive predictive formulas (intercept α and slope β).

## 3. Results

### 3.1. Measurement of Phantom Fat Concentration

The fat phantoms of varying concentrations appeared uniformly milky white, with smooth surfaces and no visible impurities or fat precipitation. Axial and coronal slices of the phantoms were scanned via the MRI-PDFF sequence (GE HealthCare Discovery MR750 FatFrac sequence). On MR images, the phantoms appeared as homogeneous gray-white structures. The proton density was measured within the ROI box and is displayed as a percentage. The ultrasound attenuation parameters of the fat phantoms were measured via three different ultrasound systems (Canon Aplio i900, Mindray Resona R9 Pro, and GE LOGIQ E11). Images acquired by different machines showed dense and bright echoes resembling those of human fatty liver. When the fat concentration changes, the echogenicity of the phantom shows no significant variation. (Figure 1) The acquisition of attenuation coefficient, backscatter, and speed of sound is shown in Table 2, which aligns with the conclusions from previous studies [19,25].

### 3.2. Quantification of MRI-PDFF

**Figure 2:**
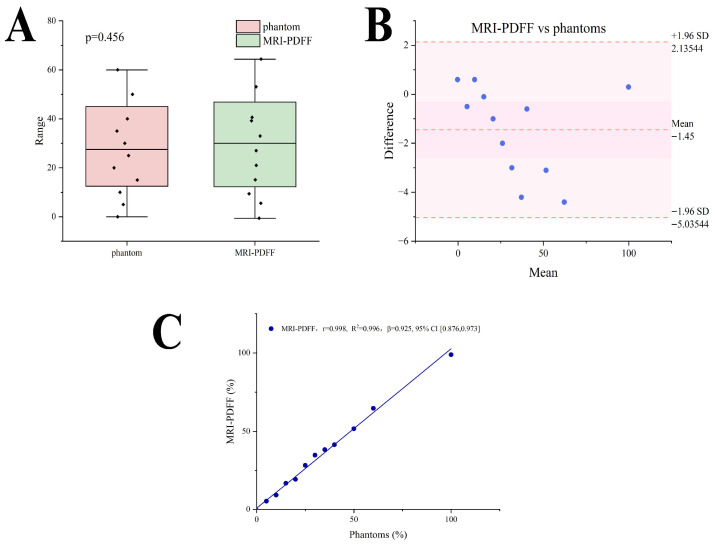
Quantification of MRI-PDFF: (**A**) the equivalence testing between MRI-PDFF and phantom fat concentrations; (**B**) Bland-Altman analysis between MRI-PDFF and phantom fat concentrations; (**C**) the linear relationship between MRI-PDFF and phantom fat concentrations.

The intraclass correlation coefficient for repeated MRI-PDFF measurements was 0.998 (95% CI, 0.996, 0.999). The equivalence testing revealed no statistically significant differences between MRI-PDFF and phantom fat concentrations (*p* > 0.05) (Figure 2A). The intraclass correlation coefficient between MRI-PDFF and phantom fat concentrations was 0.998 (95% CI, 0.993, 0.999), and Bland-Altman analysis revealed no significant deviations between the two with all scatter points within the 95% limits of agreement (Figure 2B). A significant linear correlation was found between the MRI-PDFF and phantom fat concentrations (r = 0.998, R^2^ = 0.996, β = 0.925, 95%CI [0.876,0.973], *p* < 0.001) (Figure 2C).

### 3.3. Quantification of AC

**Figure 3:**
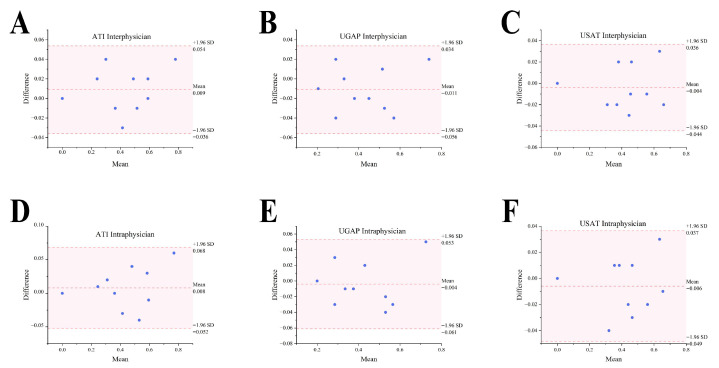
Inter- and intra-operator Bland–Altman analysis for AC measurement: (**A**) Bland–Altman analysis for inter-operator ATI measurements; (**B**) Bland–Altman analysis for inter-operator UGAP measurements; (**C**) Bland–Altman analysis for inter-operator USAT measurements; (**D**) Bland–Altman analysis for intra-operator ATI measurements; (**E**) Bland–Altman analysis for intra-operator UGAP measurements; (**F**) Bland–Altman analysis for intra-operator USAT measurements.

In terms of intra- and interphysician reproducibility, as summarized by the Consistency Analysis in Table 3, there were no statistically significant biases in the AC values obtained from the three US manufacturers by two physicians (T Zhang and J Zeng). Bland-Altman analysis revealed all scatter points within the 95% limits of agreement, and the intraclass correlation coefficient was 0.990–0.995 (Figure 3A–C). Likewise, the AC results captured by one physician (T Zhang) in different scanning planes fell within the 95% limits of agreement (LoA) (*p* > 0.05), and the intraclass correlation coefficient was 0.989–0.995, as shown in Table 4. (Figure 3D–F).

**Figure 4:**
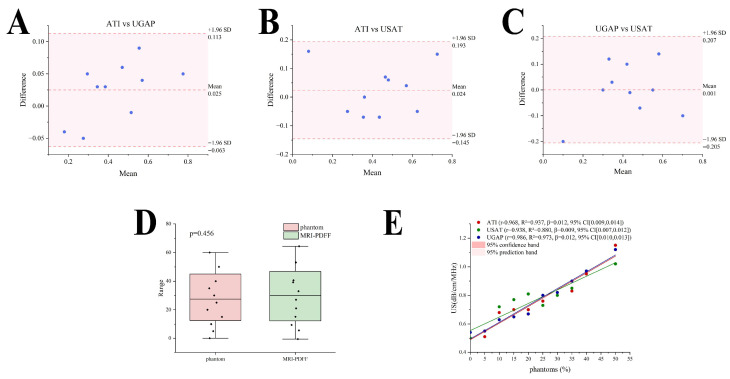
Comparison of US measurements and the linear relationship between US and MRI-PDFF: (**A**) Bland–Altman analysis between ATI and UGAP; (**B**) Bland–Altman analysis between ATI and USAT; (**C**) Bland–Altman analysis between UGAP and USAT; (**D**) the equivalence testing among the three measurement methods; (**E**) the linear relationship between US and phantom fat concentration.

The consistency analysis revealed that the interclass correlation coefficient (ICC) in the ATI data, UGAP data, and USAT data was 0.963 (95% CI, 0.890, 0.990). Table 5 shows that Bland-Altman analysis revealed no significant deviations between pairwise comparisons of the ultrasound attenuation systems, with all scatter points within the 95% limits of agreement (Figure 4A–C). The equivalence testing demonstrated no statistically significant differences among the three measurement methods (*p* > 0.05) (Figure 4D). A strong linear relationship was observed between the ultrasound attenuation parameters and phantom fat concentration, with coefficients of determination as follows: ATI (r = 0.968, R^2^ = 0.937, β = 0.012, 95%CI [0.009,0.014], *p* < 0.001), USAT (r = 0.938, R^2^ = 0.880, β = 0.009, 95%CI [0.007,0.012], *p* < 0.05), and UGAP (r = 0.986, R^2^ = 0.973, β = 0.012, 95%CI [0.010,0.013], *p* < 0.001) (Figure 4E).

### 3.4. Linear Relationship Between AC and MRI-PDFF

**Figure 5:**
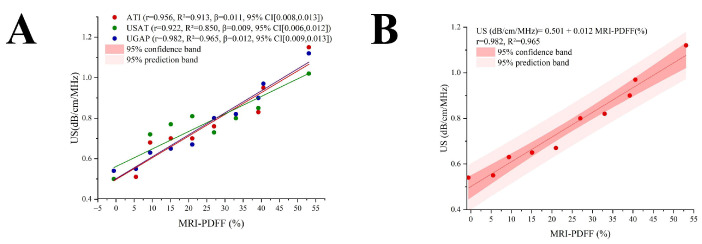
Linear relationship between AC and MRI-PDFF: (**A**) he linear relationships between US and MRI-PDFF; (**B**) the conversion formula between the ultrasound attenuation coefficient and MRI-PDFF: US (dB/cm/MHz) = 0.501 + 0.012 MRI-PDFF (%).

The following ultrasound attenuation parameters demonstrated strong linear relationships with the MRI-PDFF: ATI (r = 0.956, R^2^ = 0.913, β = 0.011, 95%CI [0.008,0.013], *p* < 0.001), USAT (r = 0.922, R^2^ = 0.850, β = 0.009, 95%CI [0.006,0.012], *p* < 0.05), and UGAP (r = 0.982, R^2^ = 0.965, β = 0.012, 95%CI [0.009,0.013], *p* < 0.001) (Figure 5A). A US-MR linear conversion formula was obtained in terms of the linear dependence between the US ACs and the MRI-PDFF values via a data scatter plot. Among the three groups, the UGAP data showed the strongest linear correlation with the MRI-PDFF measurements on the basis of the Pearson correlation coefficient (r). Therefore, the regression formula for the UGAP group and MRI-PDFF was selected as the conversion formula between the ultrasound attenuation coefficient and MRI-PDFF: US (dB/cm/MHz) = 0.501 + 0.012 MRI-PDFF (%). According to the derived linear conversion, a PDFF of 0% corresponds to 0.50 cm/dB/MHz. Other liver fat values can also be compared against this linear relationship in pairs. Notably, when the fat concentration of the phantom is greater than 50%, the ultrasonic attenuation parameter cannot be measured, so this formula is applicable only to situations where the fat concentration is less than 50% (Figure 5B).

## 4. Discussion

MASLD is currently a serious public health issue, with a prevalence rate of 25–30% in the general population [26,27]. The inability of the liver to regulate lipid homeostasis and coagulation parameters is a risk factor for atherosclerosis and cardiovascular diseases. MASLD can further progress to cirrhosis and even liver cancer, posing a significant threat to human health [28,29]. The advantages of ultrasound attenuation parameters include noninvasiveness and convenience, and they hold great potential for development in the screening, treatment, and monitoring of fatty liver disease [30].

Currently, various medical device manufacturers are developing their own ultrasound attenuation measurement systems. The diagnostic performance of ATI, USAT, and UGAP in quantifying liver fat has been evaluated in studies using MRI-PDFF and histology as references and has shown a high diagnostic accuracy and a strong correlation between AC measurements and MRI-PDFF (Dag et al., 2025; Ferraioli et al., 2019; Kuroda et al., 2021) [31,32,33]. However, there is currently no unified standard for AC measurements across different manufacturers’ systems for hepatic steatosis grading, which causes confusion in the clinical diagnosis of fatty liver disease [11]. Additionally, for human studies, the pathological gold standard for diagnosing fatty liver disease and the pathogenic sources of severe fatty liver are difficult to obtain [31]. Our study developed a kind of fat phantom for ultrasound and MRI-PDFF measurements, allowing precise control over the fat concentration of the phantom. Known objective fat concentrations can serve as standard values during different ultrasound attenuation parameter measurements, enabling a comparison and correlation between simultaneous ultrasound and MRI-PDFF measurements of the phantom. This makes ultrasound-assisted diagnosis of fatty liver disease more intuitive and straightforward.

Our experiments demonstrated that when scanning the same phantom with three different scanners and their corresponding algorithms, the measurement results from different machines and different scanners had good consistency, which was similar to the previous research [34]. This finding can help standardize the measurement criteria for ultrasound attenuation parameters in fatty liver disease.

In our study, when the phantom fat concentration was <50%, a strong linear relationship was observed between the ultrasonic attenuation parameters and the MRI-PDFF, and a linear conversion formula between the two, US (dB/cm/MHz) = 0.501 + 0.012 MRI-PDFF (%), was successfully derived. Previously, ultrasonic attenuation parameters were often represented using thresholds to indicate the severity of fatty liver disease [33,35,36]. The establishment of this formula enables the conversion of ultrasound attenuation parameters into a percentage format, thereby allowing more intuitive assessment of hepatic fat content. This advancement is expected to provide significant benefits for clinical practice.

The benefits of mapping ultrasound attenuation coefficients to the PDFF are as follows: first, it broadens the range of representations for ultrasound attenuation coefficients, making it numerically more beneficial for indicating and distinguishing the severity of the disease. Second, it establishes a correlation between ultrasound attenuation coefficients and the MRI-PDFF, facilitating their combined application in monitoring the extent of fatty liver in patients. Third, it standardizes the units and measurement criteria of ultrasound attenuation systems from different manufacturers, aiding clinicians and patients in more clearly assessing the grade of fatty liver for clinical treatment.

Our study has several limitations. First, the current study remains limited to the phantom experimental stage. The inherent heterogeneity and acoustic complexity of real liver tissue cannot be fully simulated by phantoms. Moreover, the study lacks validation across different fat concentration gradations. Further animal experiments and clinical studies are required to verify the applicability of this calculation formula. Second, our experiment utilized three ultrasound attenuation systems from three manufacturers, and many other manufacturers’ systems were not included in this study. Therefore, additional data collection and validation from more ultrasound systems of different manufacturers are needed to improve the accuracy of the conversion formula between ultrasound attenuation systems and MRI-PDFF [37,38,39]. Additionally, when the phantom fat concentration exceeds 50%, AC measurements lack stability, and the application of this formula under conditions of fat concentration > 50% remains unvalidated.

In summary, our experiments developed phantoms with varying fat concentrations and performed quantitative measurements using three AC measurement techniques: ATI, USAT, and UGAP. The phantom scans were subsequently analyzed for linear correlation with MRI-PDFF. Our results demonstrated good consistency across the three ultrasound systems, with observed linear relationships between AC values and both MRI-PDFF measurements and phantom fat concentrations, allowing for mutual conversion. On this basis, we derived a conversion formula between the two. This study provides a reference for future research on the consistency of measurements from different ultrasound attenuation systems in humans and their convertibility with MRI-PDFF, contributing to a more straightforward and comprehensible application of ultrasound technology in the diagnosis of fatty liver disease.

## Figures and Tables

**Table 1:** Ultrasonic Attenuation Scanning Parameters.

Technique	Manufacturer and Country	Software	Transducers	Unit of Measure	Probe	Frequency Range (MHz)	Scanning Depth (cm)	ROI Size (cm)	Placing Depth of ROI (cm)
Attenuation imaging (ATI)	Aplio 900, Canon, Japan	V2.4	i8CX1	dB/cm/MHz	i8CX1	1.8–6.4	0–10	2 × 4	3–7
Ultrasound attenuation imaging technology (USAT)	Resona R9 Pro, Mindray, Shenzhen, PRC	03(03.10.00)	SC6-1U	dB/cm/MHz	SC6-1U	1.2–6.0	0–10	3 × 3	5–8
Ultrasound-guided attenuation parameter (UGAP)	LOGIQ E11, GE HealthCare, USA	R3.2.1	C1-6	dB/cm/MHz	C1–6	1.5–6.0	0–10	1 × 4	4–8

**Table 2:** Attenuation coefficient, backscatter, and speed of sound.

Fat Content (%)	Attenuation Coefficient (dB/cm/MHz)	Backscatter	Speed of Sound (m/s)
0	0.54	0	1587
5	0.55	6.69	1576
10	0.63	8.40	1556
15	0.65	8.44	1534
20	0.67	8.59	1522
25	0.8	9.68	1509
30	0.82	10.12	1492
35	0.90	10.96	1485
40	0.97	11.01	1481
50	1.12	12.40	1470

**Table 3:** Consistency analysis of ultrasonic attenuation parameters measured by two sonographers.

	95% LoA (Mean Bias ± 1.96 SD) (dB/cm/MHz)	ICC
ATI	0.009 ± 0.045 (*p* = 0.244)	0.995 (95% CI, 0.978, 0.999)
UGAP	−0.011 ± 0.045 (*p* = 0.162)	0.990 (95% CI, 0.961, 0.998)
USAT	−0.004 ± 0.040 (*p* = 0.555)	0.994 (95% CI, 0.976, 0.998)

**Table 4:** Consistency analysis of ultrasonic attenuation parameters measured by one sonographer in different scanning planes.

	95% LoA (Mean Bias ± 1.96 SD) (dB/cm/MHz)	ICC
ATI	0.008 ± 0.060 (*p* = 0.433)	0.994 (95% CI, 0.982, 0.998)
UGAP	−0.004 ± 0.057 (*p* = 0.674)	0.989 (95% CI, 0.967, 0.997)
USAT	−0.006 ± 0.043 (*p* = 0.405)	0.995 (95% CI, 0.984, 0.999)

**Table 5:** Bland-Altman analysis of phantom fat concentration and ultrasonic attenuation parameters.

	95% LoA (Mean Bias ± 1.96 SD) (dB/cm/MHz)	*p*
ATI vs. UGAP	0.025 ± 0.088	0.111
ATI vs. USAT	0.024 ± 0.169	0.403
UGAP vs. USAT	0.001 ± 0.206	0.977

## Data Availability

The deidentified datasets generated and analyzed during the current study are available from the corresponding authors upon reasonable request, contingent on the submission of a scientifically rigorous proposal and the ability to perform analyses that meet the objectives outlined in the proposal.

## References

[1] Pereira E.N.G.d.S., Araujo B.P.d., Rodrigues K.L., Silvares R.R., Martins C.S.M., Flores E.E.I., Fernandes-Santos C., Daliry A. (2022). Simvastatin Improves Microcirculatory Function in Nonalcoholic Fatty Liver Disease and Downregulates Oxidative and ALE-RAGE Stress. Nutrients.

[2] Ferraioli G., Barr R.G., Berzigotti A., Sporea I., Wong V.W., Reiberger T., Karlas T., Thiele M., Cardoso A.C., Ayonrinde O.T. (2024). WFUMB Guidelines/Guidance on Liver Multiparametric Ultrasound. Part 2: Guidance on Liver Fat Quantification. Ultrasound Med. Biol..

[3] Liu J., Zhou L., An Y., Wang Y., Wang G. (2022). The atherogenic index of plasma: A novel factor more closely related to non-alcoholic fatty liver disease than other lipid parameters in adults. Front. Nutr..

[4] Hu C., Wang T., Zhuang X., Sun Q., Wang X., Lin H., Feng M., Zhang J., Cao Q., Jiang Y. (2021). Metabolic analysis of early nonalcoholic fatty liver disease in humans using liquid chromatography-mass spectrometry. J. Transl. Med..

[5] Kleiner D.E., Brunt E.M., Van Natta M., Behling C., Contos M.J., Cummings O.W., Ferrell L.D., Liu Y.C., Torbenson M.S., Unalp-Arida A. (2005). Design and validation of a histological scoring system for nonalcoholic fatty liver disease†. Hepatology.

[6] El-Badry A.M., Breitenstein S., Jochum W., Washington K., Paradis V., Rubbia-Brandt L., Puhan M.A., Slankamenac K., Graf R., Clavien P.A. (2009). Assessment of Hepatic Steatosis by Expert Pathologists: The End of a Gold Standard. Ann. Surg..

[7] Rinella M.E., Neuschwander-Tetri B.A., Siddiqui M.S., Abdelmalek M.F., Caldwell S., Barb D., Kleiner D.E., Loomba R. (2023). AASLD Practice Guidance on the clinical assessment and management of nonalcoholic fatty liver disease. Hepatology.

[8] European Association for the Study of the Liver (EASL), European Association for the Study of Diabetes (EASD), European Association for the Study of Obesity (EASO) (2024). EASL-EASD-EASO Clinical Practice Guidelines on the Management of Metabolic Dysfunction-Associated Steatotic Liver Disease (MASLD). Obes. Facts.

[9] Dasarathy S., Dasarathy J., Khiyami A., Joseph R., Lopez R., McCullough A.J. (2009). Validity of real time ultrasound in the diagnosis of hepatic steatosis: A prospective study. J. Hepatol..

[10] Bigelow T.A., Labyed Y., Mamou J., Oelze M.L. (2023). Attenuation Compensation and Estimation. Quantitative Ultrasound in Soft Tissues.

[11] Jang J.K., Choi S.H., Lee J.S., Kim S.Y., Lee S.S., Kim K.W. (2022). Accuracy of the ultrasound attenuation coefficient for the evaluation of hepatic steatosis: A systematic review and meta-analysis of prospective studies. Ultrasonography.

[12] Ferraioli G., Kumar V., Ozturk A., Nam K., de Korte C.L., Barr R.G. (2022). US Attenuation for Liver Fat Quantification: An AIUM-RSNA QIBA Pulse-Echo Quantitative Ultrasound Initiative. Radiology.

[13] Ferraioli G., Kumar V., Ozturk A., Nam K., de Korte C.L., Barr R.G. (2025). Noninvasive Quantification of Hepatic Steatosis Using Ultrasound-Derived Fat Fraction (CHESS2303): A Prospective Multicenter Study. MedComm.

[14] Madsen E.L., Zagzebski J.A., Banjavie R.A., Jutila R.E. (1978). Tissue mimicking materials for ultrasound phantoms. Med. Phys..

[15] Zhao R., Hamilton G., Brittain J.H., Reeder S.B., Hernando D. (2021). Design and evaluation of quantitative MRI phantoms to mimic the simultaneous presence of fat, iron, and fibrosis in the liver. Magn. Reson. Med..

[16] Chmarra M.K., Hansen R., Mårvik R., Langø T. (2013). Multimodal Phantom of Liver Tissue. PLoS ONE.

[17] Bush E.C., Gifford A., Coolbaugh C.L., Towse T.F., Damon B.M., Welch E.B. (2018). Fat-Water Phantoms for Magnetic Resonance Imaging Validation: A Flexible and Scalable Protocol. J. Vis. Exp..

[18] Endsley C., Ali S., Salhadar K., Woodward A., Garland S., Santelli J., Salimabad M.Z., Ren L., Yokoo T., Rosado-Mendez I.M. (2025). Lipid Microparticle-Based Phantoms Modeling Hepatic Steatosis for the Validation of Quantitative Imaging Techniques. Small Methods.

[19] Gong P., Song P., Huang C., Trzasko J., Chen S. (2019). System-Independent Ultrasound Attenuation Coefficient Estimation Using Spectra Normalization. IEEE Trans. Ultrason. Ferroelectr. Freq. Control.

[20] Liu S., Zheng X., Pan F., Bingzhen W., Chao T. (2024). Adaptive Edge-Enhanced Markov Chain Monte Carlo Method for Sound Speed Reconstruction in Ultrasound Computed Tomography. IEEE Trans. Instrum. Meas..

[21] Tada T., Iijima H., Kobayashi N., Yoshida M., Nishimura T., Kumada T., Kondo R., Yano H., Kage M., Nakano C. (2019). Usefulness of Attenuation Imaging with an Ultrasound Scanner for the Evaluation of Hepatic Steatosis. Ultrasound Med. Biol..

[22] Zhao Y., Jia M., Zhang C., Feng X., Chen J., Li Q., Zhang Y., Xu W., Dong Y., Jiang Y. (2022). Reproducibility of ultrasound-guided attenuation parameter (UGAP) to the noninvasive evaluation of hepatic steatosis. Sci. Rep..

[23] Dag N., Igci G., Yagin F.H., Hanci M.S., Kutlu R. (2024). Interobserver Reproducibility of Ultrasound Attenuation Imaging Technology in Liver Fat Quantification. J. Clin. Ultrasound.

[24] Koo T.K., Li M.Y. (2016). A Guideline of Selecting and Reporting Intraclass Correlation Coefficients for Reliability Research. J. Chiropr. Med..

[25] Ormachea J., Parker K.J. (2022). A Preliminary Study of Liver Fat Quantification Using Reported Ultrasound Speed of Sound and Attenuation Parameters. Ultrasound Med. Biol..

[26] Polyzos S.A., Kountouras J., Mantzoros C.S. (2019). Obesity and nonalcoholic fatty liver disease: From pathophysiology to therapeutics. Metabolism.

[27] Yang J.M., Sun Y., Wang M., Zhang X.L., Zhang S.J., Gao Y.S., Chen L., Wu M.Y., Zhou L., Zhou Y.M. (2019). Regulatory effect of a Chinese herbal medicine formula on non-alcoholic fatty liver disease. World J. Gastroenterol..

[28] Zhang Z., Song M., Lv Z., Guo M., Li C. (2022). Gut Microbiota Mediates Skin Ulceration Syndrome Outbreak by Readjusting Lipid Metabolism in Apostichopus japonicus. Int. J. Mol. Sci..

[29] Zhou L., Tang J., Yang X., Dong H., Xiong X., Huang J., Zhang L., Qin H., Yan S. (2020). Five Constituents in *Psoralea corylifolia* L. Attenuate Palmitic Acid-Induced Hepatocyte Injury via Inhibiting the Protein Kinase C-α/Nicotinamide-Adenine Dinucleotide Phosphate Oxidase Pathway. Front. Pharmacol..

[30] Ferraioli G., Berzigotti A., Barr R.G., Choi B.I., Cui X.W., Dong Y., Gilja O.H., Lee J.Y., Lee D.H., Moriyasu F. (2021). Quantification of Liver Fat Content with Ultrasound: A WFUMB Position Paper. Ultrasound Med. Biol..

[31] Dag N., Sarici B., Igci G., Yagin F.H., Yilmaz S., Kutlu R. (2025). Diagnostic Performance of Ultrasound-Based Liver Fat Quantification with Reference to Magnetic Resonance Imaging Proton Density Fat Fraction and Histology. J. Clin. Ultrasound.

[32] Ferraioli G., Maiocchi L., Raciti M.V., Tinelli C., De Silvestri A., Nichetti M., De Cata P., Rondanelli M., Chiovato L., Calliada F. (2019). Detection of Liver Steatosis with a Novel Ultrasound-Based Technique: A Pilot Study Using MRI-Derived Proton Density Fat Fraction as the Gold Standard. Clin. Transl. Gastroenterol..

[33] Kuroda H., Abe T., Fujiwara Y., Nagasawa T., Takikawa Y. (2021). Diagnostic accuracy of ultrasound-guided attenuation parameter as a noninvasive test for steatosis in non-alcoholic fatty liver disease. J. Med. Ultrason..

[34] Jeon S.K., Lee J.M., Joo I., Yoon J.H. (2022). Assessment of the inter-platform reproducibility of ultrasound attenuation examination in nonalcoholic fatty liver disease. Ultrasonography.

[35] Welman C.J., Saunders J., Zelesco M., Abbott S., Boardman G., Ayonrinde O.T. (2023). Hepatic steatosis: Ultrasound assessment using attenuation imaging (ATI) with liver biopsy correlation. J. Med. Imaging Radiat. Oncol..

[36] Jiang M., Zhang X., Wu X., Xu Z., Pan J., He H., Luo Y., Chen J. (2023). The diagnostic value of novel ultrasound attenuation analysis in detecting liver steatosis identified by the controlled attenuation parameter: A diagnostic accuracy study. Ann. Transl. Med..

[37] Omari E., Lee H., Varghese T. (2011). Theoretical and phantom based investigation of the impact of sound speed and backscatter variations on attenuation slope estimation. Ultrasonics.

[38] Nam K., Rosado-Mendez I.M., Rubert N.C., Madsen E.L., Zagzebski J.A., Hall T.J. (2011). Ultrasound Attenuation Measurements Using a Reference Phantom with Sound Speed Mismatch. Ultrason. Imaging.

[39] Rubert N., Varghese T. (2014). Scatterer Number Density Considerations in Reference Phantom-Based Attenuation Estimation. Ultrasound Med. Biol..

